# Understanding and optimizing brain health in HIV now: protocol for a longitudinal cohort study with multiple randomized controlled trials

**DOI:** 10.1186/s12883-016-0527-1

**Published:** 2016-01-14

**Authors:** Nancy E. Mayo, Marie-Josée Brouillette, Lesley K. Fellows, Positive Brain Health Now Investigators

**Affiliations:** Department of Medicine and School of Physical and Occupational Therapy, McGill University, Ross Pavilion R4.29, 687 Pine Ave W, Montreal, QC H3A 1A1 Canada; Division of Clinical Epidemiology and Division of Geriatrics, McGill University Health Center, Royal Victoria Hospital Site, Montreal, Canada; Department of Psychiatry, McGill University; Chronic Viral Illness Service, McGill University Health Centre, Montreal, Canada; Department of Neurology & Neurosurgery, McGill University, Montreal Neurological Institute, 3801 University St, Montreal, QC H3A 2B4 Canada

**Keywords:** Cognitive impairment, Depression, Brain disorders, HIV/AIDS, Cognitive assessment, Wilson-Cleary model

## Abstract

**Background:**

Chronic HIV infection commonly affects both cognition and mental health, even with excellent systemic viral control. The causes of compromised brain health are likely to be a multi-factorial combination of HIV-related biological factors, co-morbidities such as aging and cerebrovascular disease, and the erosion of coping skills, physical health, and social supports resulting from the strains of living with a chronic illness.

**Methods/design:**

This study aims to provide a better understanding of the relationship between cognitive complaints, depression, and objectively measured cognitive impairment in HIV, and of the key factors, whether biological or personal, which relate to these presentations and to their evolution over time. Characterization of this heterogeneity will permit more focused pathophysiological studies, and allow more targeted interventions. The project makes extensive use of Web-based research and health care delivery tools, aiming to provide cost-effective, “clinic ready” tools to improve brain health in HIV. This project has two overarching aims, reflecting our dual goals of understanding and improving brain health in HIV, focusing on cognitive impairment, its contributors and consequences.

The objectives are to contribute evidence for the validity of a brief brain health assessment, to estimate the extent to which HIV-related cognition-relevant clinical factors and patient-centered outcomes inter-relate and evolve over time, allowing identification of the mechanisms underpinning longitudinal change in brain health and to contribute evidence for the feasibility, effectiveness potential, acceptability, and underlying mechanisms of promising interventions for optimizing brain health. We adopt a cohort multiple randomized control trials design. A total of 900 participants will be characterized prospectively over a 27-month period to answer questions about the evolution of outcomes of interest. All participants will be offered basic brain health self-management information. Sub-groups will participate in pilot studies of specific, more intensive interventions to provide pragmatic evidence for feasibility, effectiveness, and comparative effectiveness.

**Discussion:**

This work will provide needed estimates of the burden, heterogeneity, evolution, and mechanisms underlying compromised brain health in HIV, and test a range of promising non-pharmacological interventions. This is an on-going study; the trials nested within this cohort that are currently recruiting participants were registered on 7 October 2015 (Clinicaltrials.gov NCT02571504 and NCT02571595).

## Background

People living with HIV worry about their memory, with good reason. As their life expectancies increase, it is becoming clear that both cognition and mental health can be affected, even with excellent systemic viral control. Although we are only beginning to understand these emerging co-morbidities, they are likely the result of multiple interacting processes. Various mechanisms have been proposed, directly or indirectly related to HIV. HIV infects cells within the central nervous system (CNS), highly active anti-retroviral therapy (HAART) varies in its CNS penetrance, providing a potential reservoir for viral replication, and local or systemic inflammation may affect brain function [1, 2]. Antiretrovirals may themselves be neurotoxic [3–5], and common co-morbidities such as cerebrovascular disease, substance abuse and hepatitis C infection can take their own toll on brain function. Finally, the experience of living with chronic infection can threaten brain health by affecting stress levels, coping, physical health, and social supports. Mood disorders can affect cognition even in otherwise healthy individuals [6], and in HIV specifically, self-reported cognitive concerns have been associated with depressive symptoms [7]. It may be that depressive symptoms and cognitive difficulties are two facets of brain dysfunction, or that depression affects cognitive performance through effects on attention or motivation [8]. This project acknowledges the potential inter-relationship between cognition and mood in HIV, addressing these together within a holistic framework of brain health.

Although the burden of poor brain health in HIV in Canada is unknown, it is likely to be high. Recent studies in other developed countries, using comprehensive neuropsychological assessment, report a prevalence of (primarily mild) HIV-associated neurocognitive disorders (HAND) of 30–50 % [9, 10]. Even higher rates have been documented in those over the age of 50, a rapidly expanding group at the frontier of existing knowledge about the combined effects of aging and longstanding HIV infection [11]. Depression is also common in HIV infection, with population-based prevalence of major depressive disorder estimated as high as 36 % [12]. Impaired cognition and depression, whether together or separately, strike patients in their productive years, and can affect medication adherence, occupational and social function, quality of life, and even accelerate mortality [4, 13–18]. Progress in understanding the heterogeneous, multi-factorial nature of compromised brain health in HIV requires careful clinical characterization, including of its evolution over time, accompanied by hypothesis-driven research focused on specific clinical phenotypes. Progress in predicting, treating and mitigating the impact of poor brain health requires better, practical clinical tools and evidence-based interventions specifically tailored for people living with HIV.

The nomenclature describing cognitive impairment, and the modalities used to measure cognition vary across clinical disciplines, hindering interdisciplinary research. Here, we have chosen to use the term cognitive deficit and its positive opposite, cognitive ability; we also distinguish between directly measured cognitive deficits (i.e. neuropsychological tests) and perceived cognitive deficits reported as symptoms (here measured using validated questionnaires). This method is broadly consistent with the requirements of the current diagnostic criteria for HAND [3]. Our view of cognition departs from current categorical diagnosis, focusing instead on cognitive ability as a “quantity” [19]. We propose that declines in cognitive ability compared to the individual’s own baseline will be the most useful trigger for intervention, and that stability or improvements are likely to be more important to the patient than whether they meet strict diagnostic thresholds. Rigid use of diagnostic categories may prevent recognition of real difficulties, and limit access to useful interventions for patients with high (but deteriorating) cognitive abilities [20, 21].

Current approaches to diagnosis of HAND rely on neuropsychological testing [3]. This is resource-intensive, with restricted availability. Front-line health care providers who must judge whom and when to refer are poorly equipped to respond to patients’ concerns about cognition: What symptoms signal difficulties that warrant further investigation or intervention? What interventions are appropriate? Are there patients who do not report symptoms who nonetheless have deficits and would benefit from assessment and treatment? We recognize that a key challenge in this area is to understand the link between what patients are saying, which is what matters to them, and what the objective tests indicate. Further, we need to better understand the relationship between different facets of brain health, address these within an interdisciplinary framework, and characterize their evolution over time. Developing better ways to measure both symptoms and signs that are feasible in everyday practice and tuned to the full range of abilities in this population is a crucial first step.

While better measurement and thorough description of the clinical phenomenology and its temporal evolution are necessary, they are not sufficient. People with HIV cannot afford to wait for researchers to fully understand the multiple factors that are likely to underpin the brain health challenges they are facing now. Research on the effects of exercise, self-management and cognitive training in healthy aging and mild cognitive impairment (MCI) shows promise in improving cognitive functions that are also commonly affected in HIV [22]. There is more than a conceptual parallel between these conditions. Subtle pathological aging changes are found in the brains of people with HIV [23]. We hypothesize that non-pharmacological interventions that have proved useful in aging and MCI will make a difference in mood, cognitive performance, and real world outcomes such as occupational functioning and quality of life in HIV. Here we describe the objectives, protocol and analytic plan for a project addressing key questions related to brain health in HIV using a cohort multiple randomized controlled trials design.

### Objectives

This project has three overarching aims reflecting the core goals of identifying, understanding and optimizing brain health in people living with HIV, focusing on cognitive ability, its measurement, contributors and consequences. The specific objectives are:

(i) To estimate the extent to which HIV-related clinical factors and patient-centered outcomes relevant to brain health and its consequences inter-relate and evolve over time and to explore the mechanisms underpinning longitudinal change in brain health;
(ii) To contribute evidence for the validity of a brief brain health assessment approach combining both patient-reported and measured cognitive deficits;
(iii) To contribute evidence for the feasibility, effectiveness potential, and acceptability of promising non-pharmacological interventions for optimizing brain health.

This is an on-going study, with external funding from a competitively awarded, peer-reviewed Team Grant from the Canadian Institutes of Health Research (TCO-125272), and institutional research ethics approval (Biomedical D Research Ethics Board protocol 13–047, McGill University Health Centre).

## Methods

### Cohort design

**Fig. 1:**
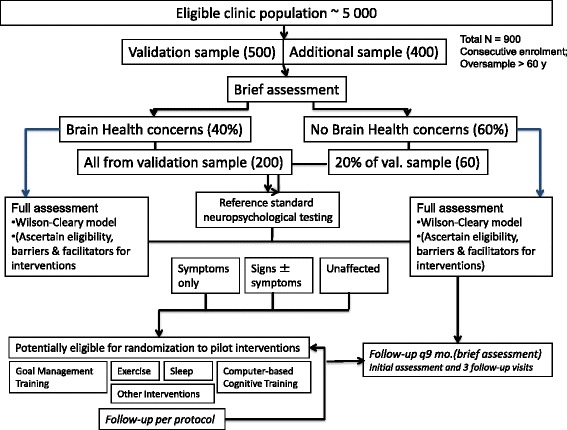
Overview of research platform, including the main cohort, followed longitudinally, and the strategy for sampling from this cohort for multiple randomized controlled trials of non-pharmacological interventions

We have developed a platform to fully characterize the heterogeneity of brain health in people with HIV, including its evolution over time and its impact. This platform also allows a sampling strategy, based on cohort multiple randomized controlled trials design [24], to identify people eligible for entering pilot studies of promising interventions. Biological specimens (serum, plasma, peripheral blood mononuclear cells, saliva for genetic analysis) are banked in anticipation of future research, including novel collaborations. The project overview is provided in Fig. 1.

### Study population

The target population is people in middle age and older, the group felt to be most at risk of both longer-term effects of HIV infection and the interaction between these and aging-related brain changes: Inclusion criteria are age ≥35, HIV+ for at least 1 year, able to communicate adequately in either French or English, and able to give written informed consent. If necessary, we will oversample patients >60 years old and women to ensure at least 100 of each are enrolled. The focus is on milder cognitive deficits, which is the area of greatest clinical uncertainty. Thus, exclusion criteria include dementia (MoCA <18) [25], or treating physician’s concern about capacity to consent, life expectancy of <3 years or other personal factor limiting the ability to participate in follow-up, non-HIV-related neurological disorder likely to affect cognition, known active CNS opportunistic infection or hepatitis C requiring Interferon-based treatment during the follow-up period, psychotic disorder, or current substance use disorder or severe substance use disorder within the past 12 months. Individuals with current or past major depressive disorder are eligible for the core project, and indeed will be the focus of one of the studies under our third aim. There are additional inclusion/exclusion criteria for individual sub-studies, described below. A brief questionnaire is used to identify characteristics of all persons approached for recruitment to determine whether non-participation introduces a selection bias into our study.

The core of the research program is the development and follow-up of a comprehensively characterized cohort of persons with HIV. The cohort is being assembled through consecutive sampling from five clinics in several large cities in Canada: Montreal, Toronto, Hamilton, and Vancouver. These clinics collectively manage thousands of patients, with diverse demographic and HIV risk profiles, most of whom are routinely followed at 3 month intervals. A total of 900 participants will be comprehensively assessed and followed longitudinally at 9 month intervals over a 3 year period (four assessments).

### Measurement strategy: the brain health platform

**Fig. 2:**
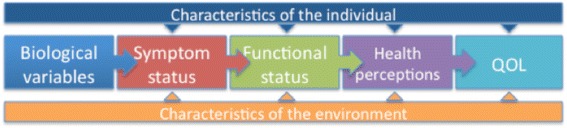
Wilson-Cleary outcome model. Characteristics of the individual include motivation, symptom amplification; characteristics of the environment include psychological and social supports

We will apply a theory-based measurement framework, the Wilson-Cleary outcome model, to structure this portion of the study (Fig. 2). This model is widely used to assess the life impact of medical conditions. The model comprehensively considers the relationship between characteristics of the individual, and of their environment, as they relate to a continuum from biological variables, to symptoms, to functional status and quality of life [26]. There have been three studies using the Wilson-Cleary model in HIV, with a restricted set of variables, and none including cognitive constructs [26–28]. Figure 2 and Table 1 present the constructs measured in the current study in all patients within the Wilson-Cleary framework. We have situated the brain health measures within the ‘symptom’ and ‘function’ rubrics of this model. Information on the key constructs is collected through chart review, face-to-face interviewing and direct testing, and through questionnaires. All questionnaires are brief and well known in the health literature, and widely tested in various populations, permitting comparisons across health conditions. To assure wide application to the broader clinical context, we selected measures that are in the public domain and available in English and French. The platform will serve as a sampling frame for a series of trials targeting brain health in selected sub-groups.

**Table 1:** Constructs to be measured within the structure of the Wilson-Cleary framework

Characteristics of the individual				
Age, sex, self-declared race, height, weight, education, work status, occupation, living situation, cognitive reserve indicators, coping, smoking, alcohol, drug use, healthy eating behaviour				
Biological and physiological variables	Symptom status	Functional status	Health perception	Quality of life (QOL)
Nadir CD4+, current immunological markers, current viral load, peak viral load, current ARV treatment, duration of HIV infection; Co-morbidities: diabetes, cardiovascular disease, hypercholesterolemia, hepatitis B & C, vitamin B12, thyroid-stimulation hormone (TSH), others Medications	Physical Symptoms: vitality, pain, sleep quality, HIV related signs and symptoms	Measured and self-reported cognitive deficits; cognitive ability; self-efficacy	General health perception; health utility	Personalized QOL; HIV-specific QOL
	Emotional symptoms: anxiety, stress, depression, apathy	Physical activity; physical function; self-efficacy for managing HIV health challenges		
		Role participation: work status, work quality, nature and frequency of leisure activities, life space mobility		
Social and psychological supports				
Social support, self-management priorities				

*ARV* anti-retroviral

### Primary outcome: cognitive ability

Measurement of cognitive ability using the B-CAM© (Brief Cognitive Ability Measure) is central to the platform. This computerized test battery was developed using Rasch Measurement Theory and Rasch analysis. Items from cognitive tests and self-report cognitive difficulties were co-calibrated to identify those that provided a measure of cognitive ability with linearized units so that the total score can legitimately be used mathematically to compare people on the same metric and to monitor change [29, 30]. Including both objective tests and self-report items is advantageous for eventual translation to routine clinical practice, in that self-report information is readily acquired and could be used to identify people in need of more in-depth assessment. The B-CAM takes less than 30 min to complete.

*Self-report Cognitive Concerns* will be measured using the Perceived Deficit Questionnaire (PDQ) [31]. This 20-item questionnaire (scored 0 to 80) measures cognitive lapses, with a score >40 indicating cognitive impairment. The PDQ has been used in several health conditions and healthy populations [32, 33].

#### Cognitive reference standard

The neuropsychological battery recently developed for CIHR Canadian HIV Trials Network (CTN) studies, the CTN Neurocognitive Battery, will serve as the reference standard for comparison purposes with B-CAM. This battery allows diagnosis of HAND as per the 2007 definition [3] by testing at least five cognitive domains with at least two tests per domain, while being as brief as possible. The tests are listed in Table 2 and take approximately 90 min to complete. In addition to diagnostic and feasibility considerations, tests were chosen to be available in both English and French with norms suitable for use in Canada.

**Table 2:** Tests Included in the Reference Standard Canadian HIV Trials Network (CTN) Neurocognitive Battery

Cognitive domains	Tasks
Memory (learning/recall)	Hopkins Verbal Learning Test-Revised (HVLT-R)
	Brief Visuospatial Memory Test-Revised (BVMT-R)
Executive functioning	Tower of London
	Stroop
	Trail Making Test B/A
Attention/working memory	Letter/Number Sequencing^a^
	Spatial Span^b^
Processing speed	Digit Symbol-Coding^a^
	Symbol Search^a^
Verbal/language	Letter Fluency FAS^c^
	Category Fluency^c^
Motor	Grooved Pegboard, dominant and non-dominant hand
IQ estimation	Toni-IV

*IQ* intelligence quotient

^a^Wechsler Adult Intelligence Scale-IV (WAIS-IV)

^b^Wechsler Memory Scale-III (WMS-III)

^c^Delis-Kaplan Executive Function System (D-KEFS)

### Multiple randomized controlled trials design

All participants are provided with an information package on strategies known to have a positive effect on brain health. Our Brain Health Now Tips cover recommendations for stopping smoking, challenging the mind, limiting alcohol and street drugs, increasing physical activity, sleeping well, managing stress and negative moods, healthy eating, and the importance of telling the medical team about all current medications. In addition to this, a series of pilot interventions targeting one or more aspects of brain health are included in this study (Table 3). Each trial has its own protocol, submitted for separate ethical review. People who meet eligibility criteria are identified from the database and those from the site where the study is conducted will be offered the intervention; eligible participants from the other sites serve as controls. Two of the trials, cognitive training and the insomnia intervention, have started recruitment. For all but two of the trials, cognitive ability is the primary outcome. The trials are designed to have a common outcome definition (responder status), analysis and sample size.

**Table 3:** Planned pilot trials for the brain health now study

Intervention	Site	Selection criteria [outcome measure]	
		B-CAM level^a^ including a self-report cognitive concern [B-CAM primary/secondary]	Other criterion
Cognitive training	Montreal	^a^[Primary]	
Insomnia intervention	All	^a^[Secondary]	Sleep dissatisfaction
Exercise	Montreal	[Primary]	Sedentary; physical function limitations
Sleep apnea intervention	Selected	[Primary]	Sleep dissatisfaction / sleep apnea screen
Goal management training	Selected	^a^[Primary]	
Cognitive behavioural therapy	Montreal	[Secondary]	Depressive symptoms
Relaxation	Montreal	[Secondary]	Anxiety

^a^indicates that selection criteria include a score lower than the mean on the B-CAM

### Data collection

A research assistant (RA) is responsible for recruitment at each of the study sites. The list of patients to be seen each day is pre-screened to identify those ineligible. Eligible patients are invited to meet with the RA who explains the study. In an observational study, it is essential to establish the extent to which the sample is representative of the target population, by collecting key information about those who decline participation, in order to understand the potential for selection bias. Therefore, those unwilling to enter the study are asked to provide a reason and respond to two questions about cognitive concerns, both of which are items from the B-CAM, and indicate age and whether they are currently working.

Those who agree to enter the study provide written, informed consent (see below). Participants next respond to the questionnaire measures directly on a clinic-based computer, guided and overseen by the study research assistant as needed, and then, if they wish, complete them at home. Data collection uses the Dacima Clinical Suite version 3 (Dacima Software Inc.) electronic data capture (EDC) software platform. The EDC platform allows data to be entered into a secure, web-based database that complies with regulatory requirement (FDA 21 CFR Part 11) through any browser. The software includes a complete audit trail, subject and data entry status tracking, real-time data validation checks, dashboards/reporting and data extraction functionalities. Data capture takes about 90 min at the first visit, and 60 min at follow-up. Site RAs assist with the administration of the B-CAM, ensure questionnaire measures are completed, and enter demographic and medical information, including standard of care blood test results abstracted from computerized clinical databases, anthropomorphic measures, patient report and chart review into the web-based data management system. The B-CAM is carried out on a separate Internet-based platform (Inquisit, Millisecond Software), and merged with the other data for further analysis.

### Statistical methods and sample size

#### Aim 1: To estimate the extent to which HIV-related clinical factors and patient-centered outcomes relevant to brain health and its consequences inter-relate and evolve over time and to explore the mechanisms underpinning longitudinal change in brain health

The analysis will estimate the complex relationships between cognitive ability, other indicators of brain health, other symptoms, such as pain, functions in everyday life, health-perception, and quality of life (QOL). We will trace the connections between biological variables, objective measures of cognition, and the constructs that really matter to patients (such as perceived health, which predicts longevity [34, 35], and QOL--the reasons for living), using the Wilson-Cleary model. The advantage of this approach is that the antecedents, correlates, and consequences of brain health can be fully described, leading to a better understanding of how to optimize function, perceived health, and QOL in the presence of lower cognitive ability.

Structural Equation Modeling (SEM), one of a family of related, sophisticated, multivariate statistical procedures, will be used to test how well the Wilson-Cleary theoretical model conforms to the data. SEM consists of two basic elements: a measurement model, analyzed by factor analysis, and a structural model, using path analysis. SEM uses latent variables to represent the constructs of interest, recognizing that complex constructs are not adequately represented by any one single measure, and thus the commonality between related measures is a better representation [36]. This method will permit the direct and indirect effects of cognitive deficits to be situated within the broader context of HIV morbidity, co-morbidities and life impact of HIV infection.

Sample size for SEM is large: optimally 15 to 20 people per parameter estimated. The number of parameters estimated in a complex model can be substantial (approximately three per included latent variable) therefore sample sizes in the range of 400 to 600 are needed for the Wilson-Cleary model. Given the focus on cognition, it will be informative to identify if the structure and relationships between and among variables differ in the presence of lower cognitive ability. As we are expecting about 40 % to have some cognitive deficit, a sample size of 900 would yield about 360 persons for an SEM model. The model will also be fit longitudinally, providing the opportunity to understand how changes in key constructs affect brain health and function over time.

To address heterogeneity of the HIV population in terms of evolution of cognitive ability over time, and co-evolution of cognitive ability with antecedent variables, correlates and consequences, a form of latent trajectory analysis (group-based trajectory analysis; GBTA) [37] will be used. This analysis is optimized with four or more time points. This approach has been used to characterize evolution of cognitive impairment over time in an elderly population [38, 39], and apathy in a stroke population [38]^5^. GBTA assumes the population is made up a mix of people with different longitudinal trajectories and uses a semi-parametric approach to group like with like. The optimal number and shape of trajectories is determined by theory, Bayesian Information Criteria (BIC), magnitude of the average posterior probability of group assignment (with ≥0.70 recommended), and closeness of the theoretical and assigned proportions of people to trajectories [37].

The sample sizes projected for this study should yield up to 5 to 7 distinct trajectories [40], providing a detailed view of the heterogeneity of longitudinal change and of factors contributing to trajectory of change. Additional multivariate approaches may be warranted, such as mixed models, to assess the impact of key variables on longitudinal change in brain health.

#### Aim 2. To contribute evidence for the validity of a brief brain health assessment approach (B-CAM)

The strength of the relationship between the results of the reference standard cognitive assessment and the B-CAM will be tested using rank correlation and sensitivity and specificity. To formally estimate the sensitivity and specificity of B-CAM, we will use the classical method of Begg and Greens [41], which assesses diagnostic tests when there are different verification probabilities. Because the new measure, B-CAM, is mapped to a standard normal distribution (on a logit scale), it is possible to use the distribution to identify a cut-point for further testing. Validation will take place on the first 500 consecutive persons enrolled. The sample size for this estimation is based on the formula provided by Begg and Greens for sensitivity, specificity and corresponding 95 % confidence intervals (CI). Assuming that approximately 40 % of people will score in a range indicative of cognitive deficit on B-CAM, we will recruit 100 % of all those with B-CAM evidence of deficit (500 * 0.4 = 200) plus 20 % of the remainder (300 * 0.2 = 60). With this verification strategy, and under the assumptions above, the estimated sensitivity is 0.86 (95 % CI: 0.73 to 0.93). The corresponding values for specificity are 0.93 (95 % CI: 0.89 to 0.96). We will also estimate the effect of covariate status (age, co-infection with hepatitis C, history of drug use, recent immigrant, or low education) on sensitivity and specificity.

#### Aim 3: to contribute evidence for the feasibility, effectiveness potential, and acceptability of interventions holding promise for optimizing brain health

The analysis for all of the intervention trial cohorts is a mix of within- and among-cohort contrasts including contrasts with those eligible for selection, but not selected, who can serve as controls. We have designed these pilots to identify the proportion with a cognitive response rather than calculating an average response and comparing averages over time or between cohorts. This responder-status analysis provides more relevant information both at a group level and at an individual level. As the time frames for each pilot may be different, the definition of a responder can reflect these differences. For example, responder-status could be defined immediately post-intervention or at follow-up or both. With a simple responder-status analysis, it is possible to calculate the probability of achieving a certain responder proportion, which will be useful for designing future trials.

For between-group comparisons, logistic regression will be the global approach to analysis with responder-status (yes-no, defined the same way for intervention and control cohorts) as outcome. The exposure is cohort: intervention vs. control. The control group is likely much larger than the intervention cohorts, so comparing each cohort to the control is statistically reasonable. People may not complete all of a given intervention, which may affect opportunity for change; a restricted analysis will be conducted on those completing at least 60 % of the sessions, recognizing that power is reduced but the information nonetheless valuable for the design of future studies.

Generalized estimating equations (GEE) will also be applied as a secondary, more general approach that permits other time points to be modeled, and consideration of other outcomes. This accommodates either binary (responder status) or continuous (scores on cognitive tests) outcomes. This analysis uses a regression model, but clustering of outcomes within time is controlled. For binary outcomes, the effect of group (intervention or control) is expressed as an odds ratio; for continuous outcomes the parameter is an effect size equivalent to an adjusted paired-*t*-test. An interaction term tests whether the effect differed by group (i.e. was larger in the intervention group, as hypothesized).

Additional analyses will be used to explain changes in cognitive status as a function of changes expected from the intervention. As the intervention cohorts are small, we will use concordance parameters, rather than a regression model, to quantify the degree to which changes in hypothesized mechanisms by which the interventions operate (improving exercise capacity, physical activity, healthy living) are concordant (at the individual level) with changes in the outcomes (cognitive ability or self-efficacy for self-management).

There were two considerations in estimating sample size. First, for the GEE analysis, a minimum sample size of 30 has been suggested [42, 43]. Second, the probability of achieving a specific response proportion can be estimated using the normal approximation to the binomial distribution when response proportions are not extreme (http://stattrek.com/online-calculator/binomial.aspx). Assuming that, in the absence of intervention, there is only a small probability of making a positive cognitive response, say 10 % (*p* = 0.10), then with 30 subjects, the probability of observing seven or more responders is unlikely, *p* < 0.03. For other outcomes (depression and anxiety) which may have a higher probability of positive response in the absence of intervention, say 20 %, then 11 or more responders out of 30 would need to be observed to be less likely than 0.05 to have occurred by chance.

### Ethics, consent and permissions

The project was approved by Research Ethics Board of each of the participating institutions, with the caveat that the pilot trials would each have their own protocol and ethics submission. All participants provide written, informed consent at the time of enrollment in the cohort. Where deemed ethically permissible, we also collect limited information from eligible individuals who refuse participation in the study in order to estimate selection bias. The local research ethics board at one center did not approve the collection of information from refusers. For the intervention studies that are recruited through telephone or email contact and are delivered only through the Internet, no additional visit is needed. We obtained ethics approval for these intervention studies only from the center from which the study was going to be conducted, after confirmation that this approach was in compliance with the national regulatory framework in Canada.

The informed consent document stipulated that any clinical results of importance for the participant’s medical care would be provided to their physician. Most blood tests are clinically indicated and are reviewed as part of routine care. For the information collected on the questionnaires, in collaboration with the site principal investigators, we developed a list of “red flags” to identify participants who may be in serious distress. The “red flags” pertain to issues of mood, pain and quality of life, as those were considered important to medical care. On a monthly basis, answers to selected questions are extracted from the database and the participants’ physicians are alerted to the presence of worrisome self-reports.

## Discussion

This novel design, cohort multiple randomized controlled trials, uses the strength of a large, epidemiologically designed, observational study to provide a representative sample that can be characterized prospectively to answer questions about the evolution of outcomes of interest. In addition, sub-groups of the sample are identified for whom pilot testing of specific personalized interventions would provide pragmatic evidence for feasibility, effectiveness, and acceptability to patients. The study will also provide a powerful platform for validating novel measurement approaches and estimates of the burden and heterogeneity of cognitive deficits over time, and will support hypothesis-driven research on mechanisms.

The findings from this work will be rigorous and the extent of their generalizability can be estimated. We expect the results will apply to similar populations cared for in specialized HIV clinics in Canada and the developed world more generally, at least. We believe this whole person approach to brain health, emphasizing evolution over time, and testing non-pharmacological interventions, will yield both conceptual advances and practical, clinically-relevant information applicable in the day-to-day management of brain health in HIV. We anticipate making this rich dataset publically available at the completion of the study, following publication of the primary research findings.

This study is also an example of interdisciplinary and multi-site team science, enabled by funding from the Canadian Institutes of Health Research that was earmarked to promote the development of new teams to address emerging co-morbidities in HIV. We have included community partners as part of our team. They have been involved from the beginning, endorsing the relevance of addressing cognitive concerns. These individuals have also been invaluable for informing methods of data collection and championing the study in the community.

Chronic HIV infection poses complex challenges that are likely exacerbated by aging and the accrual of co-morbidities, and likely affect brain health through multiple paths. This project takes a multi-pronged approach to measuring cognition and mental health concerns, understanding the basis of differences in cognitive ability, and assessing potential interventions. We apply a novel approach, the cohort multiple randomized controlled trials design. Since a pivotal paper on this method was published in 2009 [24], only a handful of studies have proposed this design [44–48]. This will be the first study to use this design to test multiple interventions.

## Abbreviations

ARV: anti-retroviral
B-CAM: brief cognitive ability measure
CD4: cluster differentiation 4
CI: confidence interval
CNS: central nervous system
D-KEFS: Delis-Kaplan executive function system
GBTA: group-based trajectory analysis
GEE: generalized estimating equations
HAART: highly active antiretroviral therapy
HAND: HIV-associated neurocognitive disorders
HIV: human immunodeficiency virus
IQ: intelligence quotient
MCI: mild cognitive impairment
MUHC: McGill University Health Centre
OR: odds ratio
PDQ: perceived deficits questionnaire
QOL: quality of life
SEM: structural equation modeling
WAIS-IV: Wechsler adult intelligence scale-IV (WAIS-IV)
WMS-III: Wechsler memory scale-III (WMS-III)
